# A Deadly Infodemic: Social Media and the Power of COVID-19 Misinformation

**DOI:** 10.2196/35552

**Published:** 2022-02-01

**Authors:** Michael A Gisondi, Rachel Barber, Jemery Samuel Faust, Ali Raja, Matthew C Strehlow, Lauren M Westafer, Michael Gottlieb

**Affiliations:** 1 The Precision Education and Assessment Research Lab Department of Emergency Medicine Stanford University Palo Alto, CA United States; 2 Stanford University Stanford, CA United States; 3 Department of Emergency Medicine Brigham and Womens Hospital Harvard University Boston, MA United States; 4 Department of Emergency Medicine Massachusetts General Hospital Harvard University Boston, MA United States; 5 Stanford Emergency Medicine International Department of Emergency Medicine Stanford University Stanford, CA United States; 6 Department of Emergency Medicine University of Massachusetts Worchester, MA United States; 7 Department of Emergency Medicine Rush University Chicago, IL United States

**Keywords:** COVID-19, social media, misinformation, disinformation, infodemic, ethics, vaccination, vaccine hesitancy, infoveillance, vaccine

## Abstract

COVID-19 is currently the third leading cause of death in the United States, and unvaccinated people continue to die in high numbers. Vaccine hesitancy and vaccine refusal are fueled by COVID-19 misinformation and disinformation on social media platforms. This online *COVID-19 infodemic* has deadly consequences. In this editorial, the authors examine the roles that social media companies play in the COVID-19 infodemic and their obligations to end it. They describe how *fake news* about the virus developed on social media and acknowledge the initially muted response by the scientific community to counteract misinformation. The authors then challenge social media companies to better mitigate the COVID-19 infodemic, describing legal and ethical imperatives to do so. They close with recommendations for better partnerships with community influencers and implementation scientists, and they provide the next steps for all readers to consider. This guest editorial accompanies the Journal of Medical Internet Research special theme issue, “Social Media, Ethics, and COVID-19 Misinformation.”

## The COVID-19 Infodemic

The COVID-19 pandemic continues to have a substantial impact worldwide, with over 266 million diagnosed cases and over 5 million deaths [1]. In 2021, depending on the month, COVID-19 was either the first, second, or third leading cause of death in the United States, alongside heart disease and cancer [2]. People are still dying from COVID-19 despite a vaccine surplus in wealthy countries, public health interventions to curb viral transmission, new therapeutic options, and the heroic efforts of frontline care providers. Why? Although we initially focused on a deadly and contagious virus, we were simultaneously overwhelmed by the deadly and contagious impact of online misinformation and disinformation about that virus [3]. Much like the COVID-19 pandemic itself, we face a widespread disease with long-term consequences: the COVID-19 infodemic.

The World Health Organization defines an infodemic as “too much information or false and misleading information” that “causes confusion, risk taking behaviors...and mistrust of health officials” [4]. The United Nations Educational, Scientific, and Cultural Organization considers *fake news* a general term for false information that can be further defined by intentionality [5]. Misinformation consists of “information that is false but not created with the intention of causing harm,” whereas disinformation is “information that is false and deliberately created to harm a person, social group, organisation, or country” often orchestrated with financial or political motives [5]. Both are prevalent across all social media platforms [6]. Together, these serve to undermine trust in governmental interventions, public health responses, expert guidance, and scientific facts about COVID-19 [7,8]. Accordingly, we define the COVID-19 infodemic as the overwhelming amount of complex and often contradictory information available about COVID-19, inclusive of substantial fake news about the origins of the virus, treatment options unsupported by rigorous clinical data, and baseless claims regarding adverse effects of lifesaving vaccines; these false narratives may be spread by authoritative institutions or influencers who are otherwise thought to be trustworthy, and they play a substantial role in shaping views and influencing human behaviors that can lead to poor health outcomes.

The clinical impact of the COVID-19 infodemic is profound. Effective strategies such as masking and social distancing have been undermined to the detriment of those at greatest risk. With several effective vaccines now available for SARS-CoV-2, vaccine hesitancy and vaccine refusal—two distinct problems with different causes and different solutions—have become major issues. Vaccine hesitancy is prolonged deliberation or delay in accepting vaccination, even when supply is ample; this differs from vaccine refusal, which is defined by the specific intent not to vaccinate, similar to the “anti-vax” movement adherents, in which people refuse all vaccines including childhood vaccinations. Both vaccine hesitancy and refusal are fueled by misinformation on social media, and vaccine misinformation that initially manifests offline can quickly spread to social media platforms; the misinformation exchange is bidirectional [9]. In fact, the US Surgeon General warned in 2021 that misinformation is the greatest threat to COVID-19 vaccination efforts [10]. COVID-19 misinformation and disinformation on social media increases vaccine hesitancy, lowers vaccination rates, and causes preventable deaths, especially among certain demographic populations [11,12]. The COVID-19 infodemic remains deadly, and we must act.

To address this, the Stanford University Ethics, Society, and Technology Hub and the Stanford Department of Emergency Medicine cosponsored INFODEMIC: A Stanford Conference on Social Media and COVID-19 Misinformation. INFODEMIC convened experts from the fields of social media, medicine, public health, and biomedical ethics with a goal of identifying new best practices to combat COVID-19 misinformation online [13]. The corresponding Journal of Medical Information Research theme issue, “Social Media, Ethics, and COVID-19 Misinformation” builds upon this work to discuss the impact of this infodemic and approaches to ending it. In this editorial, we will examine the role of social media companies (executives, financiers, leaders, and users) in health misinformation and their obligations to mitigate the COVID-19 infodemic.

## The Role of Social Media

Recognition of social media’s power to propagate fake health news came well before COVID-19, notably surrounding topics such as tobacco use, vaping, and recreational drug use [6]. However, 2020 was the *year of online disinformation*, with political and scientific misinformation and disinformation often reinforcing one another [14]. Social media companies and platform users both played a substantial role in the birth of the COVID-19 infodemic that year. The internet propagates knowledge rapidly and globally, typically without checks for accuracy, and facilitates the current infodemic. Social media companies attempt to self-police erroneous content on their platforms with variable success, both due to the overwhelming amount of COVID-19 information they must process and their reluctance to censor their users’ posts. Information filters swiftly through various avenues on the internet, often accessed via search engines and social media algorithms. Google is the dominant search engine with over 3.5 billion global searches each day, allowing individuals to retrieve information from a wide array of sources [15]. Although it may appear that users pull information, search engines prioritize certain results, in effect pushing relevant information to the user [16]. Social media algorithms push selected content to billions of users as well (Table 1) [17]. The proprietary algorithms used by social media companies are routinely exploited to spread COVID-19 misinformation and disinformation, with certain content repeatedly presented to users who have specific profiling characteristics or search histories. These algorithms could be better optimized to reduce the online trafficking of harmful information that risks the public health.

**Table 1:** Approximate numbers of monthly users of several social media platforms.

Social media platform (company, location)	Approximate number of users
Facebook (Meta Platforms, Inc; Menlo Park, California)	3 billion
YouTube (Google LLC; San Bruno, California)	2.3 billion
WhatsApp (Meta Platforms, Inc; Menlo Park, California)	2 billion
Instagram (Meta Platforms, Inc; Menlo Park, California)	1.4 billion
Twitter (Twitter; San Francisco, California)	400 million

In addition to social networking, an increasing number of users consume news on social media platforms compared with traditional media outlets [18]. Individuals engage in social circles and networks on these platforms virtually, and they do not leave—their beliefs are reinforced by information chosen for them and others like them by computer algorithms. Without intervention, there is exposure to new content but little to no exposure to new knowledge or ways of thinking. The algorithms used by social media companies create news echo chambers that can serve as a vector for misinformation by amplifying low credibility information sources. During the early COVID-19 pandemic, low credibility sources dominated both Twitter and Facebook posts related to COVID-19, topping traditional news and media outlets [19]. Online *bots* further confuse users and reduce their ability to discern truth from fake news. Bots are computer codes designed to appear as user profiles or credible news sources but are instead weapons for disinformation. Social media companies struggle to identify and remove bots that use even the simplest artificial intelligence, which take advantage of platform data and social media push algorithms. Thus, it is unsurprising that social media platforms fuel hoaxes and misinformation about the etiology and origins of COVID-19, its treatment, and its prevention through vaccines [19]. Social media companies could invest greater resources to combat these agents of the infodemic.

Moreover, health misinformation is not confined to COVID-19. In a 2021 systematic review, the greatest prevalence of health misinformation was found on Twitter and related to smoking, drugs, and vaccines [6]. Who is to blame? Many fingers can be pointed, and social media companies are among the culpable. *Top-down* misinformation from celebrities and other public figures that are allowed on these platforms exacerbates the problem. Celebrities account for 20% of online misinformation and 70% of the attention of platform users, compared to noncelebrity posts [19]. Social media companies benefit from increased user activity, and celebrity influencers are engaging. These attention-grabbing individuals enjoy unfettered reach to users because social media companies rarely place limits on their messaging, even when that messaging includes erroneous facts about COVID-19. The blurred lines between factual news and entertainment and falsehoods about COVID-19 could be labeled for users by social media companies through better oversight of their platforms.

## An Obligation to Act

US-based social media companies are legally regulated by the US Federal Trade Commission in the same ways that any other US-based businesses are regulated. However, they are not subject to federal *social media regulations* of any kind—because there are none [20]. Social media platforms are private companies who set their own internal regulatory policies and are not subject to oversight by the US Federal Communications Commission (FCC) nor, specifically, Section 230 of the Communications Act of 1934 [21]. Subsection (c) (1) of the Act maintains that social media companies do not act as publishers of information, as do other media entities, absolving them of an obligation to monitor user-generated content on their platforms. There are no legal mandates to the manner or methods by which they self-govern, and any actions by these companies are simply made in good faith. However, they do maintain internal policies, some of which are intended to curb dissemination of different types of information that are harmful to the public welfare, ranging from COVID-19 misinformation to communications among terrorist organizations. These Good Samaritan policies determine what constitutes an acceptable use of their platforms and draw a line limiting certain types of social expression [22].

That said, social media companies are mostly unregulated, and some claim they should remain so, rather than be subject to FCC oversight as are television and radio companies. Current actions by these companies are voluntary, not compulsory, and often in response to external pressures. However, if companies do not meaningfully address misinformation and disinformation on their platforms, government oversight should be considered. Internal policies are an important first step, yet we continue to see blatantly false information that contradicts scientific evidence regularly posted across most social media platforms [3]. For example, a content analysis study that evaluated 1225 fake news stories found that social media platforms were responsible for disseminating half (50.5%) of the identifiable misinformation [3]. Social media companies are partly responsible for fueling the COVID-19 infodemic, and we believe that ongoing inaction or inadequate action to address it keeps them complicit. Given the stakes, failure to address health misinformation and disinformation should be viewed as a public health crisis, and the commensurate response should include government oversight of social media companies (similar to other media sources) in the name of the public good.

Beyond law and public policy, there are other interventions to consider. We maintain that there is an ethical obligation for social media companies to act. Bioethicists recognize the broader public health consequences of social media use and how bias is determined by the specific design and implementation of social media platforms [23]. Ethical frameworks guide moral decision-making and action/inaction, two of which are especially relevant for social media companies [24]. First, utilitarian ethics calls for decisions that positively affect the greatest number of people. This is also a bedrock of public health. The application of utilitarian ethics suggests that companies should make socially conscious decisions, even when inconvenient [25]. For example, social media companies can and should redesign their algorithms that have propagated the infodemic, even if such changes risk ad revenue and profits. Similarly, censorship of celebrity users who disseminate misinformation might decrease user engagement and activity with a platform, but these actions are needed to address the infodemic. Second, virtue ethics is reasoning based on the virtues of what makes a good person, or in this case a good company. Kaptein [26] defined corporate virtue ethics that include clarity, transparency, and sanctionability, among others. A *good* social media company that earnestly engages in self-regulation would exhibit many other virtues including honesty, courage, self-control, and integrity. When considering corporate virtues, and what makes a *good* social media company, it is worth noting corporate vices in the absence of *good*: deficiency, ambiguity, subversiveness, and opaqueness [26]. These vices are commonly associated with the current practices of social media companies, specifically when considering their algorithms that repeatedly drive dangerous content to users. These algorithms reinforce COVID-19 misinformation for some users and cloister them from reports on legitimate scientific evidence.

It does not have to be this way. There is hope for “good social media companies,” ones which take bold actions in the face of a global pandemic, and perhaps at their own expense. We think that social media companies could become allies after all, especially as their power to enact social change is unprecedented. Indeed, many companies are working to mitigate misinformation already. For instance, Twitter, Facebook, and Google Search remove content, add warning labels, and deactivate misleading accounts that promote disinformation. However, these companies must navigate the substantial influx of constantly changing information about COVID-19, struggling to discern between deceptive content, scientifically inaccurate *alternative facts*, and even genuine scientific disagreement [27]. That struggle deserves consideration. For example, a claim that SARS-CoV-2 is airborne may have been flagged as misinformation or disinformation in January 2020. Today, in early 2022, most scientists believe that the virus is airborne at least in certain environments and conditions. Would a social media company that removed a post claiming that SARS-CoV-2 is airborne have harmed the public or prevented important academic debate in the early days of the pandemic?

Acknowledging the genuine complexity of the situation does not undermine but rather illuminates the urgency of our call to action. In fact, recognizing the difficulties that social media companies have in fairly adjudicating misinformation implies that far more financial and human resources will need to be marshaled to sufficiently address the problem. Wealthy companies have invested some capitol toward these efforts, but they are capable of so much more. The volume of misinformation is impressive, and removed information is often quickly replaced by similarly harmful messages within a platform, stifling progress. In essence, social media companies are treading water, at best, and additional resources and initiatives are warranted. Such efforts may need to be strategically directed toward specific aspects of the infodemic, such as misinformation about the clinical severity of the virus or disinformation about the efficacy of the vaccine. Different strategies are warranted to address a range of fixed beliefs that may have developed at different stages of the pandemic; a singular approach by a social media company may be insufficient to change minds about the origins of the virus, its transmissibility, and its prevention, all at once. Each type of misinformation deserves a unique message in response, and these messages must be tailored to the cultural differences of users in certain communities.

Addressing the infodemic does not fall solely to social media companies, and we cannot rely only on a few people or a small number of entities to battle it. Key potential change agents also include elected government representatives, public health officials, research scientists, journalists, clinical ethicists, and physicians. However, for many constituencies, a general distrust of government and an underappreciation of science is magnified in part by social media, requiring a truly multidisciplinary response to the challenge [28]. Therefore, we believe that major impact could also result from effective messaging delivered by trusted community leaders who can, for example, reach communities of vaccine hesitant individuals online and in person: ministers, youth counselors, teachers, social and mental health workers, frontline workers, and others.

Finally, implementation scientists have been inadequately used as resources. Implementation science is the study of methods used to introduce research findings into a health care context. With respect to COVID-19, the methods used to introduce a new vaccine to the entire world’s population represented an important and frequently missed opportunity [29]. During 2020, significant attention was given to the rapid development and testing of COVID-19 vaccines; relatively less attention was given to equally crucial areas, including how to equitably manufacture vaccines to scale and how to equitably distribute them [30]. The COVID-19 vaccine is widely accessible throughout most industrialized nations now, but the potential influence of implementation science remains no less important. A key tenet of implementation science is the use of different strategies to target different patient populations [31]. Especially in the face of stiffening vaccine hesitancy in certain communities and subpopulations, we believe that implementation scientists should be engaged to help guide strategies and actions of social media companies, and similarly to help other change agents such as elected officials. There is a social imperative and an ethical imperative to embrace the best available evidence and methods needed to improve COVID-19 vaccination rates. We recommend that social media companies seek the advisement of experts in implementation science as they develop strategies to combat vaccine and other COVID-19 misinformation.

## Next Steps

Health misinformation and disinformation have been increasing rapidly for over a decade [14]. During the COVID-19 pandemic, substantial attention was initially focused on ensuring the distribution of the vaccines themselves but, unfortunately, *not the distribution of reliable information nor the mitigation of harmful misinformation and disinformation.* This has had long-lasting effects. Over time, some who have said “I won’t wear a mask” now say, “I won’t take a vaccine.” Going forward, it is imperative that we move beyond our roles as scientists in a laboratory, physicians on the frontline, or strategists at social media companies. We must seek to expand our influence in health education and public health messaging more broadly. We must exit the silo of the house of medicine and meet patients and the public where they are at: online. As we do this, we need to rely on sound strategies gleaned from education and leadership literature to reach our patients effectively [8,32]. We need to identify evidence-based interventions that effectively dismantle online misinformation and then implement them [14].

If we want to create meaningful change, we cannot merely rely on the progress of clinical science alone. We must consider how best to implement and disseminate new discoveries to the public via social networks and offline communities. For many in science and medicine, this may mean engaging with mass media for the first time. That means personalizing our direct outreach to patients and communities, engaging with empathy, and seeking to understand before seeking change [33]. Moreover, we must resist a paternalistic approach in which we protect patients from information but rather empower them to seek reliable information and make informed choices about their health. Fortunately, there are frameworks that can guide us. Bautista et al [33] proposed a two-step conceptual model for physicians seeking to refute misinformation—step 1: identification of the types and sources of health misinformation; and step 2: attempting to make private and public corrections, done strategically and respectfully. Meanwhile, Chou et al [34] urged physicians to partner with social media companies and influencers to address health misinformation online, teach the public to recognize potential misinformation, and cultivate better trust toward the medical community. Finally, Walter et al [35] confirmed that interventions to correct health misinformation are the most successful when they come from experts in a given field.

Complicated problems call for collaborative approaches. Social media companies, medical professionals, researchers, implementation scientists, and trusted messengers must form synergistic partnerships to successfully combat the COVID-19 infodemic and health misinformation and disinformation generally. Rather than focusing on assigning blame, change agents can be most effective by demonstrating their willingness to act and implement new best practices, regardless of whether or not they previously contributed to some of the problems we face today.

As you read the articles in this special theme issue of the Journal of Medical Internet Research, we urge you to reflect on expanding your own contributions beyond your current working environment. In Textbox 1, we offer several actionable next steps for social media companies and health care providers to combat COVID-19 misinformation. Consider how we all can better address the current COVID-19 infodemic and combat and prevent future ones. To truly win this battle, we must urgently convert our expertise into the right words and the right actions. Whether we find our patients in the clinical environment or on social media, we must protect them from the harms of misinformation and disinformation and help them benefit from the lifesaving medical and health information that we have to offer.

**Actions to address the COVID-19 infodemic.**

**Recommendations for social media companies**

- Redesign social media algorithms to reduce the spread of COVID-19 misinformation
- Identify and remove harmful bots from platforms
- Censor sources of COVID-19 misinformation and disinformation
- Label erroneous content
- Promote sound science
- Support public health efforts
- Target culturally appropriate messaging to specific communities
- Direct users to local health clinics and COVID-19 resources

**Recommendations for health care providers**

- Engage patients on social media
- Offer COVID-19 content expertise to social media companies and online news media
- Commit to posting public health messaging online
- Identify and implement evidence-based interventions to combat health misinformation
- Partner with online influencers to disseminate accurate COVID-19 information
- Provide expert advice to mass media outlets
- Personalize direct outreach to patients and communities
- Seek to understand patients with empathy before seeking behavior change
- Empower patients to seek reliable health information and make informed choices
- Create synergistic partnerships with leaders in other disciplines

## Abbreviations

FCC: Federal Communications Commission
